# O24 - Mite allergy prevention study

**DOI:** 10.1186/2045-7022-4-S1-O24

**Published:** 2014-03-14

**Authors:** Zaraquiza Zolkipli, Graham Roberts, Ramesh Kurukulaaratchy, Louise J Michaelis, Sharon Matthews, Catherine B Clayton, Sarah Pearson, Victoria Cornelius, Syed Hasan Arshad

**Affiliations:** 1 NIHR Respiratory Biomedical Research Unit, University Hospitals Southampton NHS Foundation Trust, Southampton, United Kingdom; 2 Clinical and Experimental Sciences, Faculty of Medicine, University of Southampton, Southampton, United Kingdom; 3 David Hide Asthma and Allergy Research Centre, St Mary’s Hospital, Isle of Wight, United Kingdom; 4 Great North Children's Hospital, Newcastle, United Kingdom; 5 NIHR Biomedical Research Centre, Guy’s and St Thomas’ NHS Foundation Trust and King’s College London, London, United Kingdom

## Background

Infants with family history of atopy are considered at high risk for developing allergic disease. Environmental exposures are modifiable risk factors with a potential for intervention to prevent allergy. Children who develop house dust mite (HDM) sensitisation by pre-school age are at higher risk of developing asthma. Prevention of HDM sensitisation in early childhood by immune modulation may consequently prevent development of asthma. We sought to achieve this by inducing tolerance to HDM through high dose allergen exposure.

## Aim

We aim to provide preliminary evidence of efficacy and safety of sublingual immunotherapy with HDM in reducing the development of atopy among infants of high-risk families.

## Methodology

We conducted a randomised, double-blind, placebo-controlled trial over 2 sites: Southampton and the Isle of Wight. Infants at high risk (at least 2 first-degree relatives with atopy) were recruited at 5-6 months of age and remained on the intervention for 12 months. Exclusion criteria were skin prick test (SPT) positivity to screening food and aeroallergens, prematurity and concurrent major health problems. Participants underwent clinical and immunological assessments at baseline. The intervention was sublingual immunotherapy of either house dust mite extract or saline (ALK-Abello, Denmark). Assessment, including SPT was performed every 3 months to monitor development of allergic symptoms and SPT positivity (atopic sensitisation).

## Results

We recruited a total of 111 participants. At baseline 42% had objective evidence of eczema, 29% had parental report of wheeze and 20% had parental report of food allergy. To date, 66 infants have completed the study. Of these, 14 showed atopic sensitisation to at least 1 allergen (cumulative rate 21%). There have been no adverse reactions directly related to the interventional product.

## Conclusion

We have demonstrated the safety of sublingual immunotherapy using HDM in high-risk infants with a cumulative rate of atopic sensitisation of 21% over 12 months.

